# Supplementary material for: Lactic Acid Resistance and Population Structure of Escherichia coli from Meat Processing Environment
Source: Microbiol Spectr. 2022 Oct 4;10(5):e01352-22. doi: 10.1128/spectrum.01352-22 (PMC9602453; doi:10.1128/spectrum.01352-22)
Supplement: Supplemental file 2 — Fig. S1 and S2. Download spectrum.01352-22-s0002.pdf, PDF file, 0.2 MB [file spectrum.01352-22-s0002.pdf]

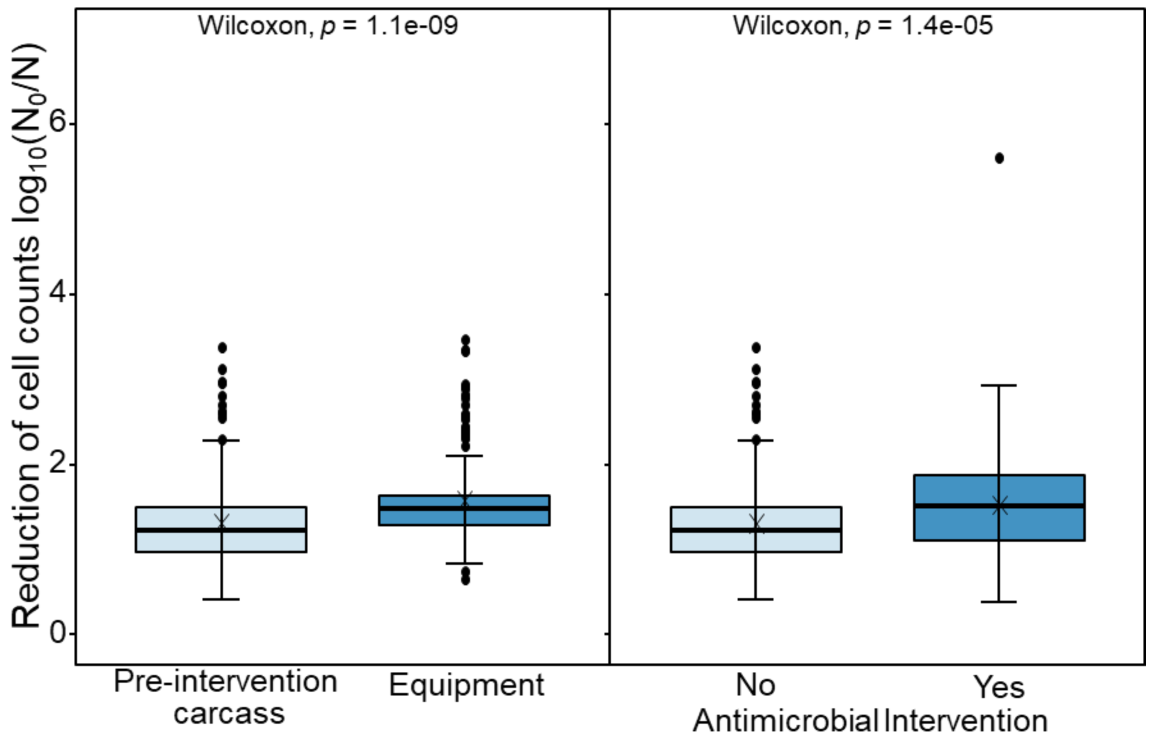

Figure S1. Acid susceptibility of *E. coli* associated with carcasses prior to intervention (pre-intervention carcasses; n=200) or equipment surfaces (n=200; **A**), and of *E. coli* from beef carcasses pre- (n=200) and post-antimicrobial interventions (n=200; **B**) in beef processing plants. Data were replotted from data shown in Figure 1 to better illustrate the comparison between these pairs better. Pre-intervention carcass-associated *E. coli* isolates were referred to as those recovered from hide-on/dressed carcasses before any antimicrobial interventions were applied; equipment-associated *E. coli* were referred to the isolates recovered from equipment surfaces regardless of whether equipment sanitation was involved. The median log reduction is indicated by a line; the mean log_{10} reduction is indicated by ×; outliers are indicated by the black dots. The differences between the two groups in each panel were determined by the Wilcoxon test.

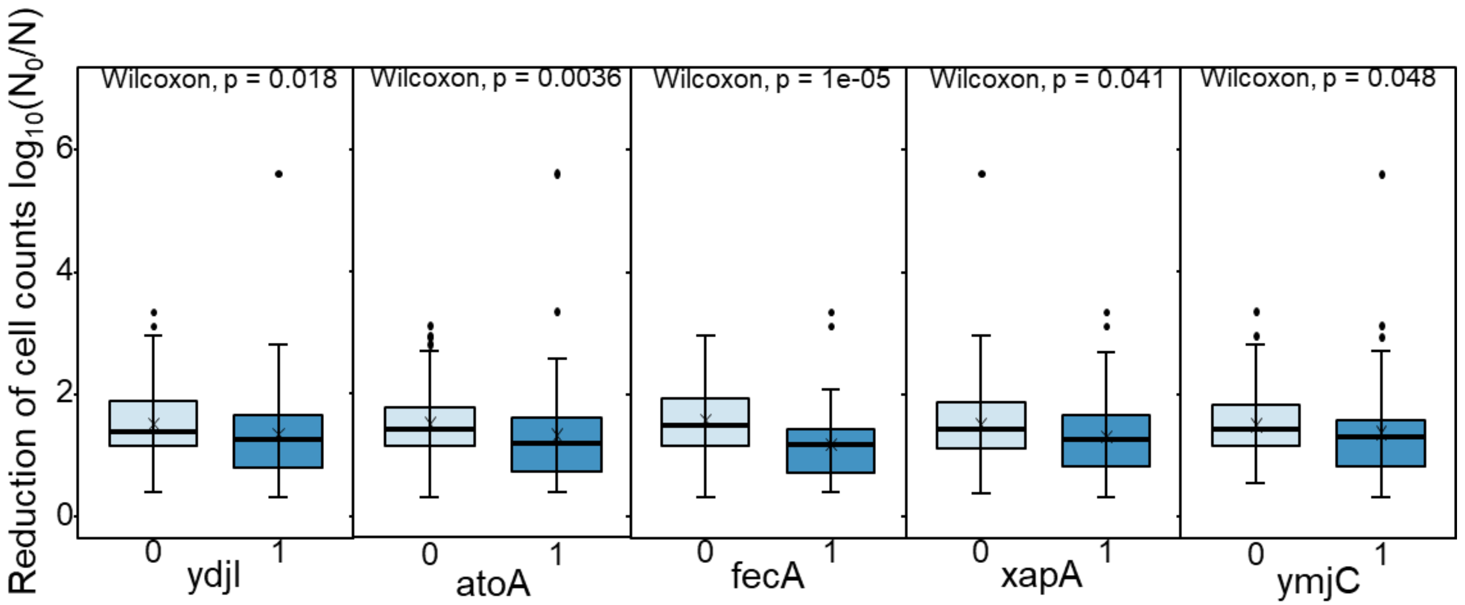

Figure S2. Reduction of cell counts by lactic acid treatment of the *E. coli* populations with (dark-blue) or without (light-blue) the genes *ydjI*, *atoA*, *fecA*, *xapA*, or *ymjC*. Data were replotted from Figure 1 to better illustrate the effect of gene presence and absence on acid resistance. The median log reduction is indicated by a line; the mean log reduction is indicated by ×; outliers are indicated by the black dots. The differences between the two groups in each panel were determined by the Wilcoxon test.
